# Surfaces with Adjustable Features—Effective and Durable Materials for Water Desalination

**DOI:** 10.3390/ijms222111743

**Published:** 2021-10-29

**Authors:** Samer Al-Gharabli, Ziad Abu El-Rub, Eyad Hamad, Wojciech Kujawski, Zuzanna Flanc, Katarzyna Pianka, Joanna Kujawa

**Affiliations:** 1 Pharmaceutical and Chemical Engineering Department, German Jordanian University, Amman 11180, Jordan; ziad.abuelrub@gju.edu.jo; 2 Biomedical Engineering Department, German Jordanian University, Amman 11180, Jordan; eyad.hamad@gju.edu.jo; 3 Faculty of Chemistry, Nicolaus Copernicus University in Toruń, 7 Gagarina Street, 87-100 Toruń, Poland; wkujawski@umk.pl (W.K.); 296530@stud.umk.pl (Z.F.); 301112@stud.umk.pl (K.P.)

**Keywords:** PVDF, roughness and chemistry tuning, desalination, membrane distillation, wettability, material chemistry

## Abstract

Materials based on PVDF with desirable and controllable features were successfully developed. The chemistry and roughness were adjusted to produce membranes with improved transport and separation properties. Membranes were activated using the novel piranha approach to generate OH-rich surfaces, and finally furnished with epoxy and long-alkyl moieties via stable covalent attachment. The comprehensive materials characterization provided a broad spectrum of data, including morphology, textural, thermal properties, and wettability features. The defined materials were tested in the air-gap membrane distillation process for desalination, and improvement compared with pristine PVDF was observed. An outstanding behavior was found for the PVDF sample equipped with long-alkyl chains. The generated membrane showed an enhancement in the transport of 58–62% compared to pristine. A relatively high contact angle of 148° was achieved with a 560 nm roughness, producing a highly hydrophobic material. On the other hand, it was possible to tone the hydrophobicity and significantly reduce adhesion work. All materials were highly stable during the long-lasting separation process and were characterized by excellent effectiveness in water desalination.

## 1. Introduction

The increasing global demand for freshwater alongside the simultaneous decline of its sources poses a threat to the sustainable growth of human societies. This threat has been a turning point for increasing efforts to convert seawater into fresh and drinkable water [1,2]. Among many available desalination methods, membrane distillation (MD) has been receiving growing attention, owing to the lower required pressure than in reverse osmosis, as well as lower operating temperature than that in the classical distillation process [3]. Membrane distillation is a technique where a porous hydrophobic membrane allows solvent vapors to be transported across the membrane. The process is thermally driven by a partial vapor pressure difference. Many modes of MD are available, and among them, the air-gap membrane distillation (AGMD) is the most interesting one. Permeate vapors are condensed on the cooled part, and then removed outside the module [4,5]. The ideal membrane material is required to be not only hydrophobic and porous to prevent wetting, but also thermally and chemically stable, and durable in long-term operations. For that reason, more efforts have been made to produce highly effective materials e.g., superhydrophobic nanofibrous membranes with the electrospinning method for MD purpose [6,7,8], polymeric membranes by different modifications, including the interference in the structure and composition by co-casting or lamination to produce finally dual-layer membranes, e.g., Janus materials [9,10,11,12]. These treatments have allowed the modification of both the roughness and chemistry of the materials. In the field of material science and membranes, poly(vinylidene) fluoride (PVDF) has been found to be very effective. In particular, it has been broadly used in MD [13], filtration [14], boron removal [15], as a separator for lithium-ion batteries [16], in biofuels recovery [17], and in water pollutants clearance [18,19]. Although PVDF is applicable and suitable for the MD process, it is not a superhydrophobic or very rough material. These features might limit the utilization in long-lasting applications when the risk of wetting could occur. For that reason, modification to the PVDF must be introduced, either by manipulating the chemistry, the roughness, or both. An interesting approach has been presented by Liu et al. [20], who made Janus materials based on PVDF with hydrophobic and hydrophilic features possessing additional clay particles to enhance roughness. The novelty was the use of ε-caprolactam as a solvent for polymeric dope preparation and to ensure the lack of delamination between layers. The membranes were highly effective in desalination accomplished via direct contact-MD at feed/permeate temperatures equal to 60 °C and 17.5 °C, respectively. The value of permeate flux for 3.5 wt.% NaCl solution was 85.1 kg m^−2^ h^−1^ [20]. Qing et al. [1,21] proposed solvent-thermal induced roughening (STIR) method to grow the surface roughness of PVDF membranes. As a result, the roughness factor (R_a_) increased from 3 to 28 nm, with a significant rise of contact angle from 132° to 155°. The same research group combined a STIR with the utilization of various types of alcohols (2-propanol, 1-butanol, 1-pentanol, 1-heptanol, 1-decanol) during the process [1]. It was reported that, depending on the size of molecules and affinity between PVDF and alcohols, the adjustment of the fraction distribution of crystal α (nonpolar) and β (polar) phases in the PVDF membrane was possible. The most interesting results and anti-wetting features were achieved for the 1-pentanol-treated PVDF membrane. The material was characterized by a water contact angle of 164.1°, sliding angle of 8.1°, roughness parameter R_a_ of 15 nm, and water flux in the DCMD of 21 kg m^−2^ h^−1^.

The effect of pore morphology and surface roughness on the wettability of porous titania films was presented by Xiong and co-workers [22]. Based on the collected data, it was stated that surface wettability presents that pore morphology and surface roughness could substantially impact the wettability. They highlighted the valuable meaning of adjusting material properties of the membranes [22].

**Figure 1:**
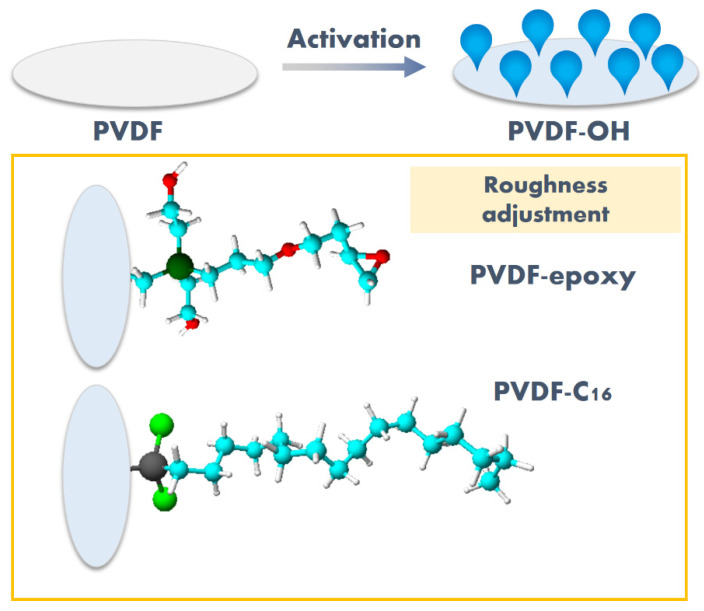
The idea of the work—functionalization of activated (PVDF-OH) materials to tune material properties. PVDF-epoxy—membrane modified with 3-glycidyloxypropyl)triethoxysilane, PVDF-C_16_—membrane modified by trichloro(octadecyl)silane.

Guided by the literature survey, in this work, the excellent features of the PVDF were employed to tune the chemistry and morphology of targeted membranes. Hence, the focus was on activating PVDF membranes based on the method developed previously by our group [13,23] to carry out a functionalization process. The impact of chemistry and roughness modifications were assessed on the membrane performance in the desalination process via MD as demonstrated in Figure 1. For that reason, the modifiers with different characters and length/size of molecules were selected i.e., 3-glycidyloxypropyl)triethoxysilane, and trichloro(octadecyl)silane. The element of novelty is to apply modifiers with varied structure, reactive groups—reactivity to tune chemistry and roughness, particularly when a grafting agent with a very long chain is applied. Due to the formation of PVDF surface enriched in hydroxyl groups, all modification were accomplished via the chemical route.

## 2. Results and Discussion

### 2.1. Impact on Morphology

**Figure 2:**
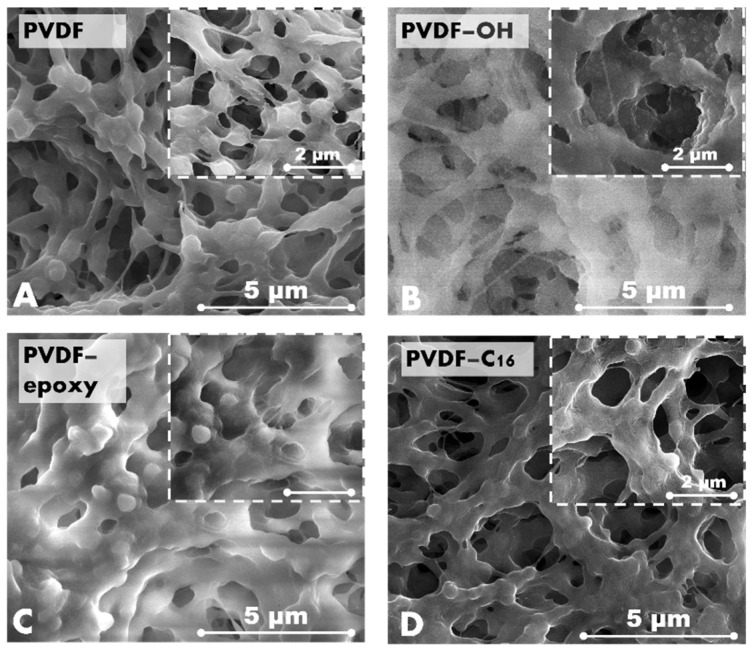
SEM images of pristine (**A**), activated (**B**), and modified samples, PVDF-epoxy (**C**), PVDF-C_16_ (**D**).

The activation process of the polymer, as well as all types of modifications, significantly affected the membrane morphology (Figure 2). The first step of the treatment, i.e., piranha activation, generates a blister-like structure due to its oxidative features. This piranha effect was observed in our previous works, when the procedure was established [13,23]. The second step of the process, i.e., functionalization leading to the changes of the morphology with clear differences, depends on the type of modifier. The materials functionalized with epoxy and C_16_ molecules possessed microroughness on the entire structure (Figure 2). The microstructure was more visible on the C_16_-treated membranes (Figure 2D). The impact of membrane modification on morphology has been presented by different research groups [24,25,26]. For the material tuned with an epoxy ring modifier, SEM analysis revealed that the material becomes denser, which can be attributed to the presence of epoxy rings as well as better coverage of the surface with more covering molecules. Such changes observable for the samples treated with short-chains modifier can be explained by occurred partially cross-linked layer, detected with MAS NMR study (Figure S1) near the surface. As a consequence of this phenomenon, changes in morphology, roughness, and physicochemistry might be observed. This behavior, in the case of membrane functionalization, has been reported in the scientific literature [13,22].

**Figure 3:**
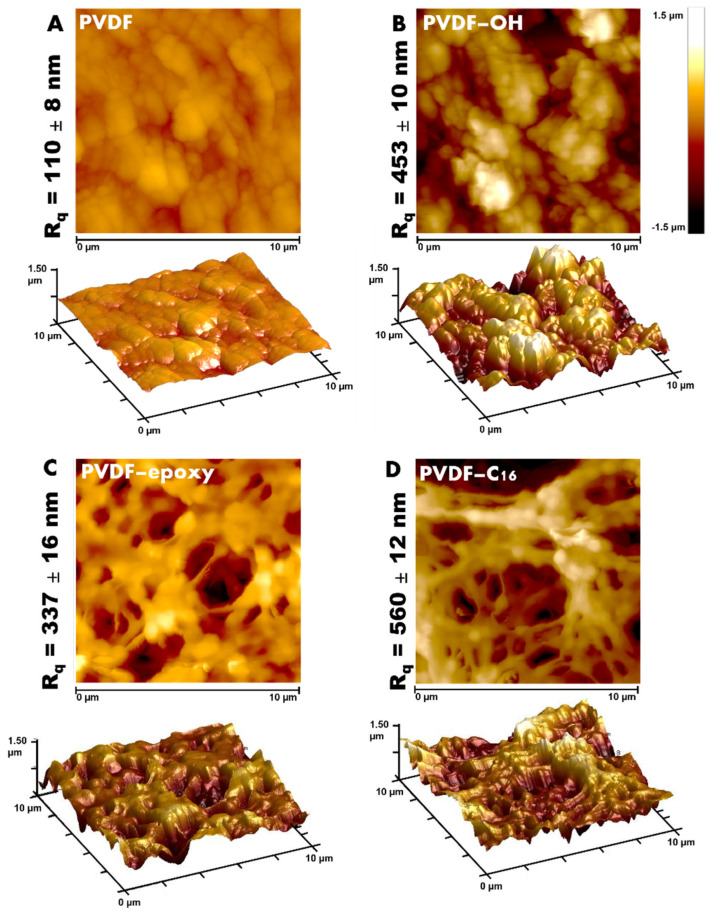
AFM—2D and 3D images of evaluated membranes, pristine PVDF (**A**), activated (**B**), with epoxy modifier (**C**), and C16 modifier (**D**).

In membrane science, changes in morphology must be evaluated from the surface level of the material as well as the entire porous structure. Therefore, pore size analysis (Figure S2, Table 1) and roughness studies (Figure 3) were performed. It can be seen that the activation process enlarged the pore size of the membrane materials. The activated membrane possessed an average pore size higher by 35.5% compared to the pristine membrane (Table 1). However, the subsequently functionalized membranes were characterized by slightly smaller pores when referring to the activated membrane (PVDF-OH). The reason for such increase in the pore size of the activated sample is related to the strong oxidative character of piranha treatment that is “digesting” the membrane materials. Nevertheless, it was already presented that in the case of utilizing the diluted aqueous solution of activator it is possible to adjust and control the process. As a conversance of the treatment, it is also possible to notice mass reduction in the samples, even up to 5%. These observations and detailed study were presented in previous work from our research group [22].

Data from pore size analyses were coherent with the observation from SEM images. The attachment of molecules with an epoxy ring caused a visible pore size reduction due to the bulkier ring than the alkyl chain, as observed in the SEM and pore size analysis (Figure 2, Table 1). A more significant pore size reduction and improvement in the roughness were found for the sample treated with C_16_. The reason for these changes needs to be sought in the utilization of a much longer chain modifier. That feature of the C_16_ molecules brought the elasticity as a consequence of surface coverage.

Although the data collected from SEM and porosimetry analyses are consistent, the AFM technique was also applied to measure the roughness parameters to better understand the differences in the surface morphology. As a consequence of the piranha treatment, an increase in the roughness factor from 111 ± 8 nm to 560 ± 12 nm was observed for all samples (Table 1, Figure 3). For the PVDF-epoxy membrane, the roughness (Table 1) was smaller than for samples treated with molecules having sixteen carbon atoms (Table 1).

### 2.2. Impact on Material Structure

PVDF possesses five polymorphs in its crystalline structure α, β, ϒ, δ, and ε [27]. The most applicable and common polymorphs in the PVDF structure are α and β. The β phase contains Trans–Trans (TT) with the orthorhombic unit cell that can be generated by applying the nucleating effect of a suitable nanofiller in PVDF matrix [28]. α phase is a Trans–Gauche–Trans–Gauche (TGTG) chain conformation possessing a monoclinic unit cell and can be formed by crystallization from melt [28]. The presence of various crystalline structures in PVDF is because of the similar atomic radii of fluorine and hydrogen in monomer units which results in chain polarization [28].

Any differences that occurred because of the modification were evaluated using a variety of spectroscopic methods. XRD revealed that the activation process increased the α-phase level of the PVDF and reduced the β-phase, causing a significant rise in the hydrophobicity of samples (Figures S3 and S4). The pristine membrane possessed major crystalline peaks at 2θ equal to 16.8° (100), 18.8° (020), 20.3° (110), and 26.9° (022), attributed to the crystal planes related to the α-phase of PVDF. The crystallinity degree expressed as a ratio of β/α [29] for the pristine material was 0.37, and 0.30 after activation. However, for functionalization using various molecules, the ratio of β/α changed as follows: 0.27 for PVDF-C_16_ and 0.28 for PVDF-epoxy.

The PVDF phases, i.e., α-phase and β-phase, contain TGTG and TT conformation chains, bringing a hydrophobic and hydrophilic character, respectively (Figure S4). For these PVDF phases, the value of total energies of conformation chains is −23.95 kJ mol^−1^ for TT (hydrophilic β) and −25.21 kJ mol^−1^ for TGTG (hydrophobic α) [30]. The value shows that the TGTG conformation is more stable than the TT one [31]. The β/α reduction in silane-modified materials confirmed the improvement in their hydrophobic character.

**Figure 4:**
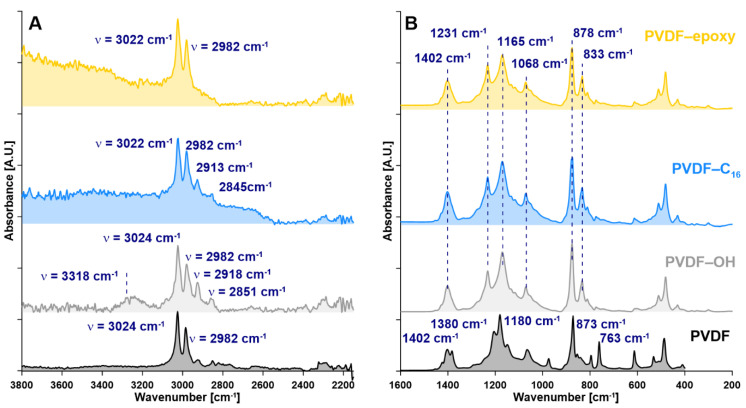
FTIR-ATR spectra of the investigated samples, (**A**)—spectra in the range of 2200–3800 cm^−1^, (**B**)—spectra in the range of 200–1600 cm^−1^.

After acquiring the knowledge of how the support has been changed by the XRD analysis, in the next step of the work, the subtle differences in materials chemistry were studied. The collected FTIR-ATR spectra (Figure 4) confirmed the high effectiveness of the activation (PVDF-OH) due to the presence of typical bands for hydroxyl groups ca. 3318 cm^−1^, as well as the bands around wavenumber of 2851, 2918, 2982, and 3024 cm^−1^. The latter ones were associated with the activation and defluorination of the PVDF polymeric membrane and then with the replacement of a single C-F bond with a single C-OH bond. A more detailed description and band assignment are presented elsewhere [13,23]. In the case of the membrane modified with C_16_, the characteristic bands were observable at 2845 cm^−1^ as well as 1016, 950 cm^−1^ in the dactyloscopic region. The successful modification of PVDF-epoxy was proven by the presence of the characteristic bands at ca. 910 cm^−1^ from epoxy groups (Figure 4).

### 2.3. Impact on Thermal Properties

It is important to assess the thermal stability of novel materials that are formed. Based on the collected data (Figure S5), it was possible to confirm that modified samples possessed better thermal stability than the pristine PVDF (T_d_ = 472.7 °C). The thermal improvement for all modified materials was comparable, and the decomposition temperature rose by ca. 20 °C. Nevertheless, all modified materials were stable in the high-temperature range. It should be emphasized that the operating temperatures of the separation process are much lower than the decomposition temperature of the membranes, which ensures high stability during the MD process (Figure S5).

### 2.4. Impact on Wettability

**Figure 5:**
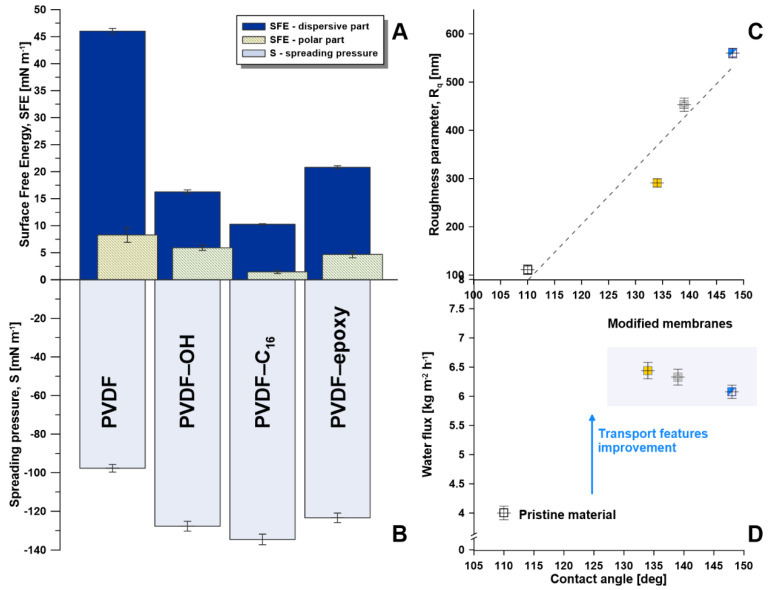
Wetting characterization, (**A**)—SFE, (**B**)—spreading pressure, (**C**)—relation between roughness and water contact angle, (**D**)—relation between water permeate flux and water contact angle.

A comprehensive wettability study was carried out, and the following factors were determined: contact angle (CA), surface free energy (SFE), spreading pressure (S) [32], liquid entry pressure (LEP), and critical surface tension (γ_cr_). Based on the XRD analysis (Figure S3), the improvement of the hydrophobic character is described as an effect of increasing the PVDF α-form. A significant reduction in the SFE of the membrane was observed (Figure 5A) for all of the modified materials, principally in the polar part of SFE (from 8.3 ± 0.08 mN m^−1^ for PVDF to 1.50 ± 0.05 mN m^−1^ for PVDF-C_16_). Surface free energy (SFE) is an essential physical feature governing polymers’ wettability, adhesive strength, and rupture phenomena. SFE plays a significant role in surface-active media producing an adsorption-induced reduction in strength to deformation (the Rebinder’s effect) [33,34]. Since SFE was calculated based on the values of contact angle corrected by the roughness [35], it was possible to exclude the impact of the roughness/irregularities that might be a source of error in SFE calculation.

The established data proved that all membranes were efficiently activated and functionalized (Figure 5). A substantial alteration between the pristine and modified materials was associated with the base and acid interactions, based on the Van Oss-Chaudhury-Good theory [36]. The higher level of basicity caused by the base piranha utilization promoted lower SFE (Figure 5A) [37,38,39,40]. In Figure 5B the spreading pressure comparison is shown. Negative values of S ensure that the material is not wettable [41]. The pristine membrane possessed the S value equal to −97.7 ± 1.9 mN m^−1^. According to the S factor, the most resistant to wetting was the membrane functionalized with C_16_ modifiers with S equal to −134.5 ± 2.7 mN m^−1^ (PVDF-C_16_). On the other hand, the epoxy functionalized membrane possessed a less negative S value due to its lower hydrophobicity (CA = 134°), −123.4 ± 2.5 mN m^−1^ (PVDF-epoxy). Nevertheless, the membranes were stable (Figure S6 —long-term stability), resistant to wetting (Figure 5C,D), and suitable for the membrane distillation process. Taking into account the method of modification, a linear relation between water flux and roughness of the modified samples was observed (Figure S7). Fluoropolymers generally possess low surface energy, which may be linked to the high electronegative feature of fluorine. Therefore, its attractive force to other substances is weak [42]. Because of its fluorinated composition, the PVDF film, by nature, displays high water contact angle values in the range of 85°–130°, which confirms its inherent hydrophobicity [43].

In the presented work, the values of the water contact angle varied between 110° (PVDF) and 148° (PVDF-C_16_). The changes in the hydrophobicity were related not only to the introduction of silanes-based molecules and the development of higher heterogeneity, but also the higher level formation of the alpha form of PVDF. A much stronger influence of the functionalization was found for PVDF-C16 due to the longest alkyl chain and chlorine at-oms as reactive groups. The chlorine group is the most labile and reactive, owing to the low value of their bonds dissociation (Si-Cl), equal to 253 kJ mol^−1^, referring to that of Si-O(Me/Et)—equal to 452 kJ mol^−1^ [44]. Due to the higher reactivity, higher hydrophobicity and roughness was observed. A linear relationship has been also found between water contact angle and roughness (Figure 5C). The established data of contact angle for water, roughness, and their relationship are in accordance with the Cassie−Baxter’s wetting model [45,46], predicting that, for an irregular hydrophobic surface, a nonwetting liquid may not enter into surface cavities, resulting in the air pockets forming a composite solid−liquid−air interface where roughness of surface upsurges with the hydrophobicity. The presented behavior was confirmed by the linear relationship between CA and R_q_ for all modified samples.

The measured critical surface tension (γ_cr_) for the pristine material PVDF was 32.3 ± 1.1 mN m^−1^, which agrees with estimates in the literature ranging between 30.3–37.4 mN m^−1^ [47,48]. Critical surface tension is an important factor in membrane science, defining the limit of material wettability, and selecting mixtures for membrane cleaning [49,50,51]. The sample will be wetted with all liquids having liquid tension less than γ_cr_ of the material [52,53]. The modified membranes were characterized by reduced values of γ_cr_ compared to the pristine ones that also ensured the improved resistance to wetting of the functionalized materials (Table 2). The most resistant was the PVDF-C_16_ membrane, which also had the highest value of liquid entry pressure, i.e., 161.24 kPa. Liao et al. [54] made PVDF nanofiber membranes for direct contact MD, which possess superhydrophobic character (contact angle = 158°) and a LEP of 146 kPa. The same research group developed PVDF composite nanofiber membranes bioinspired by lotus leaves, with robust superhydrophobicity, which were applied for MD. The materials were characterized by a LEP of 179 kPa and a contact angle of 154° [55]. Zhu and co-workers introduced dual-bioinspired membranes with superhydrophobicity for direct contact MD that was generated by the addition of negatively charged SiO_2_ nanoparticles to the polymeric dope [56]. The LEP value was quite small (95.2 kPa). Deka et al. [57] presented an interesting development of omniphobic PVDF membranes filled with zinc oxide for the oily wastewater desalination process. The membranes were characterized by a very high roughness of 1.37 µm and a contact angle of 159° with an LEP of 189 kPa [58].

### 2.5. Impact on Mechanical Features

Improving mechanical features of membranes can lead to better performance, mechanical strength increase, and tackle the membrane breakage problem [59,60]. This enhancement can be achieved in various ways, the best of which is material modification either by adding nanomaterials to the matrix or adjusting the membrane’s surface features. In the presented work, different routes for material modification were implemented. An improvement in mechanical properties was observed in all cases of membrane treatment. The highest impact was noticed in the adhesive features (F_a_) for the material treated with epoxy modifier (PVDF-epoxy) due to the introduction of more hydrophilic moieties (Figure 5, Table 2). These data were in good accordance with the results gathered from goniometric analysis, particularly from surface free energy, showing a significant rise in the dispersive component (Figure 5A). A significant effect can be observed when considering the hardness and Young modulus of the generated membranes. In the case of hardness, the improvement was in the range of 68 and 75% between the activated membrane (PVDF-OH) and the PVDF-epoxy one. Generally, the modification process had a small influence on the Young modulus, where an improvement was in the range of 6–10% only.

It must be emphasized that there was a big advantage in the presented work, which stemmed from the fact that all modifications were made via a chemical route that ensured high stability and durability of the membrane during the separation processes. The chemical anchoring of the grafting agents amended the features of the material, generating highly robust materials.

### 2.6. Transport and Separation in Membrane Distillation

Based on the gathered data from the systematic material study, it was confirmed that the generated separation materials are suitable for the MD process. The prepared membranes fulfilled all the requirements of suitable material for MD, i.e., the materials are porous, hydrophobic, and no wetting behavior occurred. In the first step of membrane application, the transport features of water across the membrane were determined. Finally, the membranes and their stability were assessed in the desalination process.

#### 2.6.1. Water Transport

Prior to the separation process, the transport properties in contact with pure water were determined. The following factors were taken into account: overall transport coefficient (Equation (S1)), permeance (Equation (S2)), and LEP (Equation (S3)) were calculated (Table 2 and Table 3). The membranes after the activation process and modification were much more permeable. The improvement in water flux was in the range of 51–62%, referring to the pristine PVDF membrane. The best enhancement of transport features (62%) was observed after modification with epoxy-equipped molecules. The water flux during the MD changed from 4.00 kg m^−2^ h^−1^ to 6.44 kg m^−2^ h^−1^ (Figure S6). Nevertheless, all membranes were very stable even during the long-lasting process, and no wetting occurred, which is crucial for membrane applicability in the MD process. During the first few hours of operation for all tested membranes, the system needed time to reach a state and subsequently maintain a stable flux. Long-term stability was determined during ca. 60 days of testing. It can be summarized that the separation materials were stable without any negative effect on the transport properties.

When the hydraulic pressure of the entering feed is higher than the LEP value, the membrane material with a hydrophobic character is wetted, and the transport of vapors cannot continue. Such a problem will exclude the membrane from further utilization in the MD process. Based on the collected data, it can be noticed that all membranes possessed positive LEP, and the modified membranes showed higher LEP values compared to the pristine ones (Table 2).

Considering other parameters, i.e., the overall mass transfer coefficient (K) and permeability, it was confirmed that the modifications improved transport across membranes. The biggest improvement was noticed for both activated (PVDF-OH) and modified with epoxy (PVDF-epoxy) membranes. However, the smallest impact was observed for the membrane functionalized by C_16_ molecules, due to its highly hydrophobic character, high LEP, and limited transport (Table 2 and Table 3).

#### 2.6.2. Desalination

**Figure 6:**
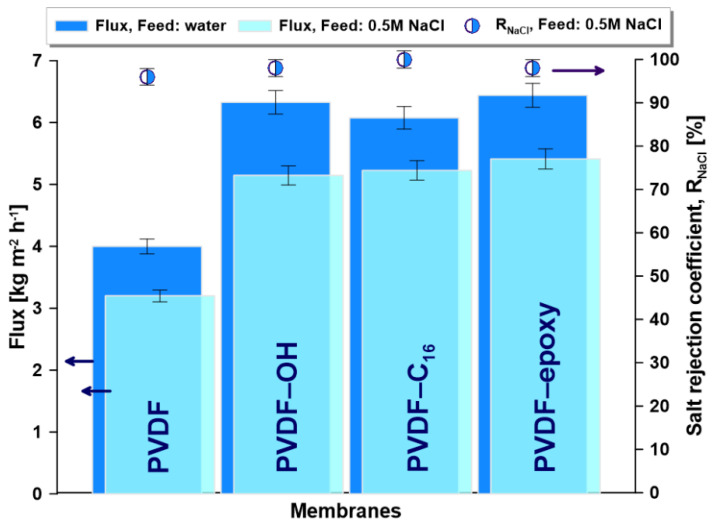
Membrane performance in the desalination process of AGMD.

In this step of MD, the membranes were assessed upon contact with salty water (0.5 M NaCl—transport and separation). Generally, during the desalination process, a diminution of all fluxes was observed because of a reduction in driving forces associated with the presence of non-volatile NaCl in the feed. It is coherent with the physiochemistry of the desalination process governed by Raoult’s law [61]. In MD, the salt rejection coefficient is a crucial parameter describing the effectiveness of separation. The salt rejection coefficients of all tested membranes were close to unity, ensuring that there was no leakage in the course of the MD process (Figure 6). The smaller marginal value of R_NaCl_ for the pristine PVDF can be linked to slight alterations in the material features (CA, R_q_, F_a_, γ_cr,_ and S). All modified membranes possessed enhanced flux in comparison to pristine. The permeate flux for pristine PVDF was equal to 3.2 kg m^−2^ h^−1^ and for the modified ones, the values were in the range of 5.1 and 5.4 kg m^−2^ h^−1^ (Figure 6). All separation materials were stable in the course of long-term MD tests (Figure S6B).

### 2.7. Stability

**Figure 7:**
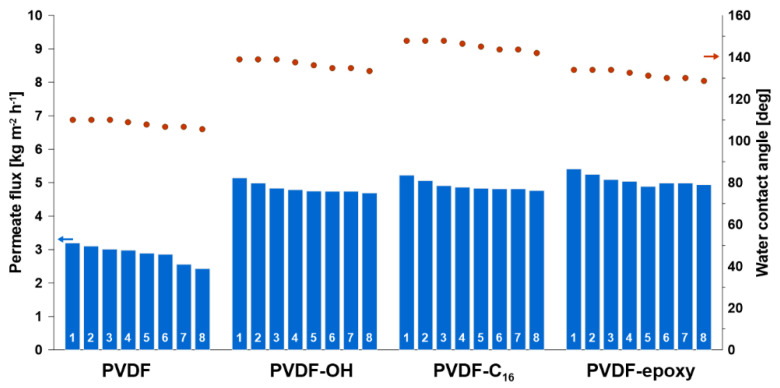
Membrane stability in desalination process—transport and material properties.

Due to the great importance of membrane stability, as well as its applicability, the membrane performance in the MD process was monitored during the eight runs per membrane, where each run lasted ca. 45 h. The membrane chemistry was evaluated in the course of the tests by measuring the contact angle. On the other hand, the membrane utility in MD was checked by measurements of permeate flux and salt rejection coefficient (Figure 7). All materials were very stable. Nevertheless, small differences were observed between the pristine and modified membranes, with the latter being more beneficial. After the last run, the reduction in transport properties was ca. 23% for the pristine material and ca. 10% for the modified materials. However, the values of the contact angle changed only 3–8%.

## 3. Materials and Methods

### 3.1. Materials

Polymeric membranes made from polyvinylidene fluoride with a pore size of 0.45 μm were purchased from Sigma Aldrich (Hamburg, Germany). All solvents were purchased from Avantor Performance Materials (Gliwice, Poland), i.e., hydrogen peroxide 30%, ammonium hydroxide 30%, methanol dichloromethane (DCM), sodium chloride (NaCl). Grafting agents, (3-glycidyloxypropyl)triethoxysilane (epoxy) and trichloro(octadecyl)silane (C_16_) were purchased from ABCR Chemical (Karlsruhe, Germany).

### 3.2. Membrane Modification Protocol

#### 3.2.1. Activation Process

To activate the PVDF materials, base piranha solution was used according to the developed procedure described elsewhere [23].

In brief, 20% water solution of 3:1 ration of ammonia and hydrogen peroxide were mixed together. A freshly prepared activator was used each time. The membrane sample, with a round shape and diameter of 47 mm, was previously wetted in methanol, then placed in the Schott glass reagent bottles to perform the procedure precisely and accurately. Finally, 15 mL of the activator was added. The activation hydroxylation process was performed for 15 min at 60 °C. Subsequently, the samples were immersed in deionized water (15 MΩ·cm) for 5 min and washed to remove any traces of activator. Furthermore, membranes were rinsed with methanol five times and dried at 70 °C overnight. Membranes labeled as PVDF-OH were used for further functionalization.

#### 3.2.2. Material Functionalization

The modification of the hydroxyl-furnished materials has been accomplished to tune the hydrophobicity/hydrophilicity, roughness, or both factors. To do so, and to adjust the properties of the material, (3-glycidyloxypropyl)triethoxysilane and trichloro(octadecyl)silane were applied. The final material was assigned as PVDF-epoxy and PVDF-C_16_, respectively. The modification step was performed for all samples under an ambient atmosphere of argon by immersing the PVDF-OH membrane in 0.1 M of the corresponding grafting agent for 3 h at room temperature. Subsequently, membranes were dried at 70 °C overnight.

### 3.3. Membrane Characterization

The following methods were implemented to evaluate the impact of the material modification on the chemistry and roughness tuning, and consequently on the transport and separation. Changes in morphology were detected by scanning electron microscope (SEM) technique applying Quantax 200 with an XFlash 4010 detector (Bruker AXS machine). Samples were sputtered with a nanolayer (of 1 nm thickness) of Au before analysis to improve conductivity and imaging quality. Changes in pore size and pore size distribution were analyzed using the Coulter Porometer II (Coulter Electronics Ltd., Luton, Bedfordshire, UK) [62,63].

Differences in surface heterogeneity were evaluated by atomic force microscope (AFM), Nanosurf Flex-Axiom microscope (Nanosurf, Liestal, Switzerland). Topography analysis was performed using AFM contact mode and ContAl-G probe with spring constants (k) of 0.2 N m^−1^ (Nanosurf, Liestal, Switzerland). Roughness parameters were determined from a maximum area of 100 × 100 µm applying Gwyddion 2.55 software. Each membrane was tested at least three times and averaged. Mechanical properties of the membranes were monitored in the nanoscale by a nano-indentation technique using the AFM machine. During the analysis, Young’s modulus (E), adhesive forces (F_a_), and nanohardness (H) were measured. The diamond probe with pyramid geometry (k = 859 N m^−1^) was selected to determine H and E. Samples were tested at least five times. As a result, an average value with ± 3% accuracy was presented. Adhesion force was analyzed with silicon nitride probes NP-1 (k = 0.58 N m^−1^). Data provided from at least 20 measurements were averaged. All AFM tests were done at ambient temperature.

ATR-FTIR was applied to analyze the changes in material chemistry after modification processes. A Bruker Vertex 80v ATR-FTIR machine was applied, and the spectra with 256 scans were collected at a resolution of 4 cm^−1^.

Thermal stability of the membranes was determined by thermogravimetric analysis (TGA) applying Jupiter STA 449 F5 from Netzsch (Selb, Germany) under ambient nitrogen atmosphere at a temperature range of 25–1100 °C with a heating rate of 20 °C/min.

Static contact angle measurements were accomplished on the Theta Flex, Biolin Scientific (Goteborg, Sweden). The results of apparent contact angle (CA) with different testing liquids (3 μL drop volume) allowed the calculation of spreading pressure (S), surface free energy (SFE), and critical surface tension (γ_cr_) [32]. The values of CA were determined with the roughness correction applying the Fringe Projection Phase-Shifting method [35]. To calculate the SFE according to the Owens, Wendt, Rabel, and Kaelble (OWRK) method, liquids with liquid surface tension in the range of 18.5 mN m^−1^ for hexane to 72.7 mN m^−1^ for water were used (Equation (S4)). The critical surface tension (γ_cr_) was determined by applying the Zisman approach [64,65].

To control the method of grafting molecules attachment, solid-state cross-polarization/magic angle spinning nuclear magnetic resonance (CP/MAS NMR) measurements for ^29^Si were implemented using Bruker Avance 700 MHz.

### 3.4. Desalination Process

All prepared and well-characterized membranes were subsequently tested in air-gap membrane distillation for desalination. Measurements were performed under a driving force of 160 ± 0.7 mbar. The difference in vapor pressure was generated by applying different temperatures to the feed and cooling parts, equal to 57 ± 2 °C and 8 ± 2 °C, respectively. Each run was performed for at least 10 h. However, it took ca. 70 h to characterize the material in the long-term stability experiment. Initially, the flux of pure water and then 0.5 M solution of sodium chloride was analyzed. The salt rejection coefficient (R_NaCl_) was evaluated using conductivity meter Elmetron CPC-505 (Poland). C_p_ and C_f_ in Equation S5 refer to salt concentration in the permeate and the feed, respectively. Water transport (Equation (S2)) was described with the implementation of various factors, i.e., overall mass transfer coefficient (K) (Equation (S6)), permeance coefficient (p_i_/L) (Equations (S3) and (S4)), and liquid entry pressure (LEP (Equation (S7))).

## 4. Conclusions

To enhance desalination processes based on membrane distillation, a new type of PVDF membranes were developed. Pristine membranes were chemically functionalized with a variety of moieties to influence surface chemistry and morphology. Membranes were activated using the novel base piranha solution and further furnished with alkyl and epoxy moieties. The produced membranes show higher mechanical, thermal, and chemical stability, as well as improved transport features. An outstanding behavior was noticed for the functionalized membranes. Based on the developed method, it was possible to design membranes with targeted material properties. It is evident that the activation process, as well as all types of modifications, significantly affected the membrane morphology. The modified membranes showed an enhancement in the transport at least of 52% compared to pristine. A relatively high contact angle of 148° was achieved with a 530 nm roughness producing highly hydrophobic material. Additionally, water flux was directly and linearly proportional with roughness for the functionalized materials. On the other hand, tuning only chemistry by introducing fluorinated alkyl chains leads to high hydrophobicity with a low polar component of SFE and significantly reduced adhesion. However, transport properties were improved only moderately. The long-term stability of fluxes in MD experiments was confirmed.

## Figures and Tables

**Table 1:** Minimum (Min), maximum (Max), and average (Aver) pore size of the investigated membranes with changes relative to the pristine and activated membranes.

Sample	Pore Size [µm]			Effect of Activation—Difference Referred to PVDF [%]	Effect of Functionalization—Difference Referred to PVDF-OH [%]
	Min	Max	Aver		
PVDF	0.429	0.662	0.551	-	-
PVDF-OH	0.571	0.837	0.747	+35.5	-
PVDF-epoxy	0.515	0.755	0.703	+27.6	−5.9
PVDF-C_16_	0.530	0.782	0.681	+23.6	−8.8

**Table 2:** Wetting (LEP, γ_cr_) and mechanical (F_a_, E, H) features of the investigated samples.

Sample	LEP [kPa]	γ_cr_ [mN m^−1^]	F_a_ [nN]	E [GPa]	H [GPa]
PVDF	74.40	32.3 ± 1.1	24.8 ± 1.1	2.11 ± 0.06	0.132 ± 0.010
PVDF-OH	129.84	28.3 ± 1.0	35.0 ± 1.1	2.23 ± 0.06	0.222 ± 0.013
PVDF-C_16_	161.24	25.4 ± 1.1	31.7 ± 1.0	2.33 ± 0.06	0.231 ± 0.016
PVDF-epoxy	132.49	27.6 ± 1.1	36.2 ± 1.2	2.33 ± 0.06	0.224 ± 0.013

**Table 3:** Overall mass transfer coefficient and permeability of the investigated membranes.

Sample	(p_i_/L) [kg m^−2^ h^−1^ bar^−1^]	K [kg m^−2^ s^−1^ Pa^−1^]
PVDF	27.23	0.75 × 10^−8^
PVDF-OH	43.06	1.20 × 10^−8^
PVDF-C_16_	41.36	1.15 × 10^−8^
PVDF-epoxy	43.84	1.22 × 10^−8^

## Supplementary Materials

The following are available online at https://www.mdpi.com/article/10.3390/ijms222111743/s1.
