# Supplementary material for: Surfaces with Adjustable Features—Effective and Durable Materials for Water Desalination
Source: Int J Mol Sci. 2021 Oct 29;22(21):11743. doi: 10.3390/ijms222111743 (PMC8583984; doi:10.3390/ijms222111743)
Supplement: Supplementary file 1 [file ijms-22-11743-s001.zip › ijms-1430500-supplementary.pdf]

Supporting information

Surfaces with adjustable features–effective and durable materials for desalination

Samer Al-Gharabli [1,*], Ziad Abu El-Rub [1], Eyad Hamad [2], Wojciech Kujawski [3], Zuzanna Flanc [3], Katarzyna Pianka [3] and Joanna Kujawa [3,*]

[1] Pharmaceutical and Chemical Engineering Department, German Jordanian University, Amman 11180, Jordan; ziad.abuelrub@gju.edu.jo
[2] Biomedical Engineering Department, German Jordanian University, Amman 11180, Jordan; eyad.hamad@gju.edu.jo
[3] Faculty of Chemistry, Nicolaus Copernicus University in Toruń, 7 Gagarina Street, 87-100 Toruń, Poland;
* Correspondence: samer.gharabli@gju.edu.jo (S.A.-G.); joanna.kujawa@umk.pl (J.K.); Tel.: +962-6-429-4404 (S.A.-G.); +48-56-611-43-15 (J.K.); Fax: +962-6-429-4404 (S.A.-G.); +48-56-611-45-26 (J.K.)

The idea of dividing the SFE into individual components includes the assumption that γsl is determined by various interfacial interactions that depend on the properties of both the measured substrate and the measurement liquid. The most often applied method for polymeric materials so far is the Owens, Wendt, Rabel, and Kaelble (OWRK) method. The method is also suitable for the coatings, aluminum, and varnishes. The benefit of the OWRK method is a fact that uses geometric mean to treat the molecular interactions which is better justified than the harmonic mean used by Wu [1-9]. The calculation of SFE and its components polar and dispersive was done according to the S1 equation. Due to the $\gamma_{sv}{}^{d}$ and $\gamma_{sv}{}^{p}$ are unknowned, two liquids with the known dispersive and polar components were applied.

$$\sqrt{\gamma_{sv}^{d}\gamma_{lv}^{d}} + \sqrt{\gamma_{sv}^{p}\gamma_{lv}^{p}} = 0.5\gamma_{lv}(1 + cos\theta_{Y}) \qquad /S1/$$

Where: γ_{sv}^{d} – dispersive part of interfacial tension between solid and vapour, γ_{lv}^{d} – dispersive part of interfacial tension between liquid and vapour, γ_{sv}^{p} – polar part of interfacial tension between solid and vapour, γ_{lv}^{p} – polar part of interfacial tension between liquid and vapour, γ_{lv} interfacial tension between liquid and vapour, $\cos\theta_{Y}$ – cosine of contact angle.

The salt rejection coefficient (R_{NaCl}) was evaluated using conductivity meter Elmetron CPC-505 (Poland). C_p and C_f in Eq.S1 refer to salt concentration in the permeate and the feed, respectively. Water transport (Eq.S2) was described with the implementation of various factors, i.e. overall mass transfer coefficient (K) (Eq. S3), permeance coefficient (p_i/l) (Eq. S4,5), and liquid entry pressure (LEP (Eq. S6).

$$R_{NaCl=}\left(1-\frac{C_p}{C_f}\right)\cdot 100\ [\%] \qquad /S2/$$

Solvent vapors transport (i.e., water) in MD can be calculated employing the following parameters (Eqs. 2 and 3) [10]:

$$J_{H2O} = K(p_f - p_p) \qquad /S3/$$

$$K = \left[\frac{1}{K_f} + \frac{1}{K_m} + \frac{1}{K_p}\right]^{-1} \qquad /S4/$$

where: J_{H2O} – water flux, K – overall mass transfer coefficient [kg $m^{-2}s^{-1}Pa^{-1}$], p_f – partial pressure of water vapor in feed, p_p – partial pressure of water vapor in permeate, K_f – coefficient of mass transfer for feed layer, K_m – coefficient of mass transfer for membrane, and K_p – coefficient of mass transfer for permeate layer.

The K factor is related to the membrane properties, e.g., morphology, porosity, tortuosity, pore size, and materials [11]. While K is also associated with pressure and

temperature, it is often a nearly constant value [11]. Bulk's equation (Eq. 4) was applied to determine the water vapor pressure as it is more precise than the Antoine's equation [12].

$$P = 0.61121 \exp\left[\left(18.678 - \frac{T}{234.5}\right)\left(\frac{T}{257.14+T}\right)\right] \qquad /S5/$$

where temperature (T) is in [°C], and pressure (P) is in [kPa].

The permeance crosswise the membranes can be established according to the flux by normalizing it to the active surface membrane area and the difference in vapor pressure across the membrane [13]. The permeability of the bulk membrane material describing pure water transport can be obtained from the permeance multiplied by the thickness of the membrane. Nevertheless, based on Baker's approach, the membrane permeance (p_i/l) can be determined even for unknown membrane thickness [14] (Eq. 5). In this case, a component flux normalized for driving force can be implemented, accordingly.

$$\frac{p_i}{l} = \frac{J_i}{p_{i,0}-p_{i,l}} \qquad /S6/$$

Where: J_i – permeate flux, $p_{i,0}$ and $p_{i,l}$ are the partial pressures of the component on the side of the membrane.

To evaluate the wettability, not only contact angle but also liquid entry pressure (LEP) was used according to the Cantor–Laplace equation (Eq. 6) [15, 16] and applying data from goniometric and porosimeter experiments.

$$LEP = -\frac{2\gamma}{r_{max}} \cos(CA)[kPa] \qquad /S7/$$

where: γ is the liquid surface tension (water at 25 °C, 71.99 mN m^{-1}), cos(CA) is the cosine of the contact angle between testing liquid and membrane, r_{max} is maximum membrane pore radius (porosimetry – bubble point) [17], pores considered to have a constant radius of curvature and have perfectly cylindrical geometry.

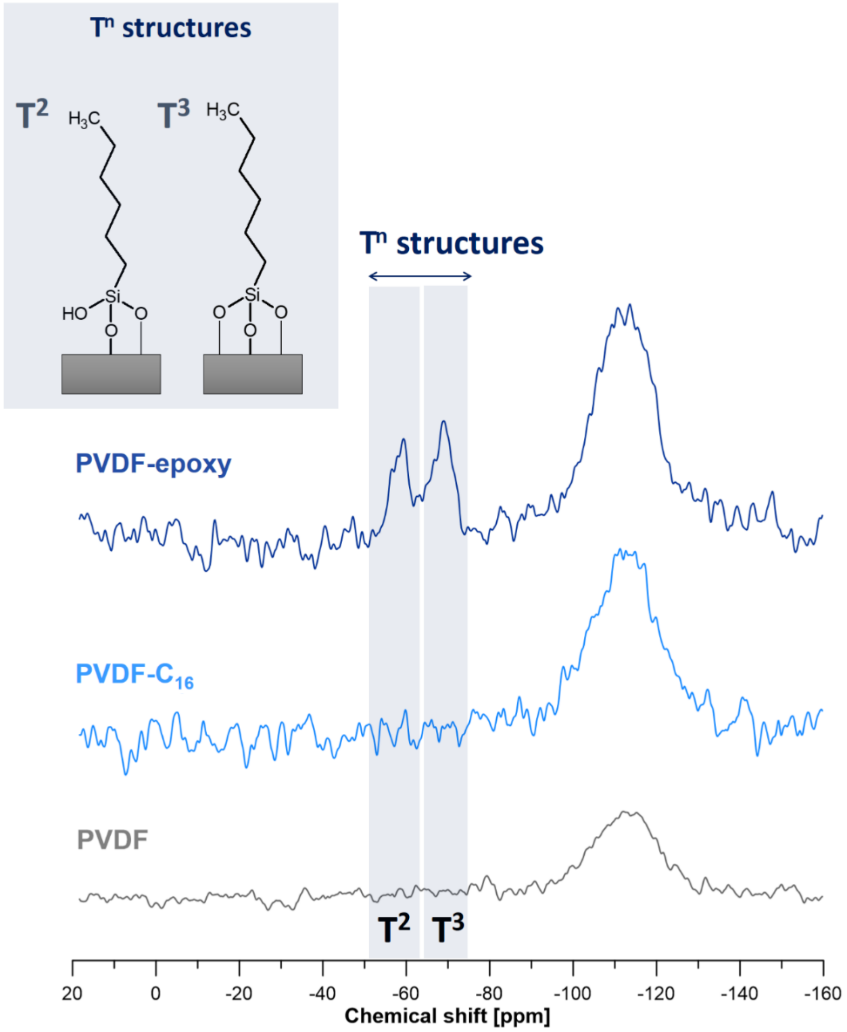

Figure S1. Solid state ^{29}Si NMR of pristine (PVDF) and modified samples PVDF-C_{16}, PVDF-epoxy.

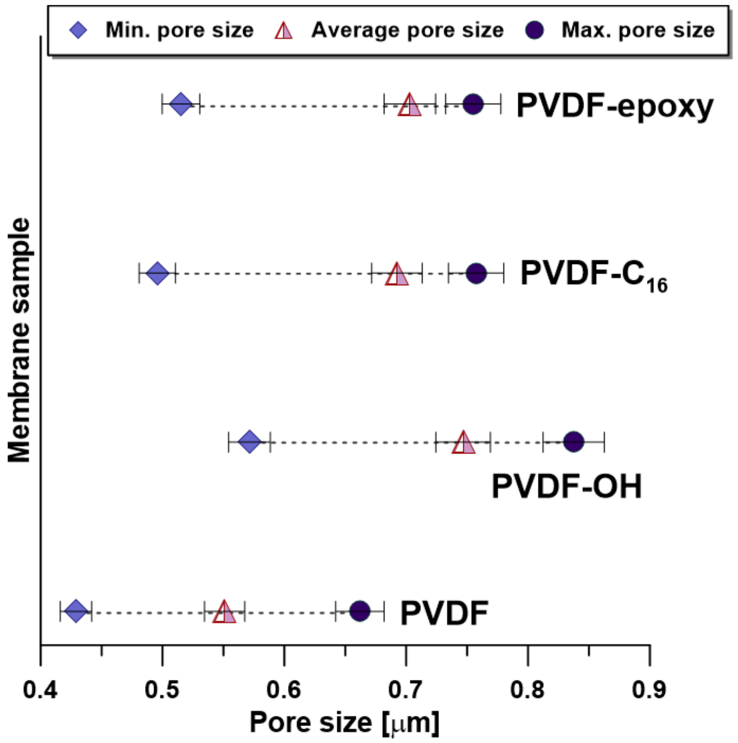

Figure S2. Pore size (minimum, maximum and average) of the investigated membranes.

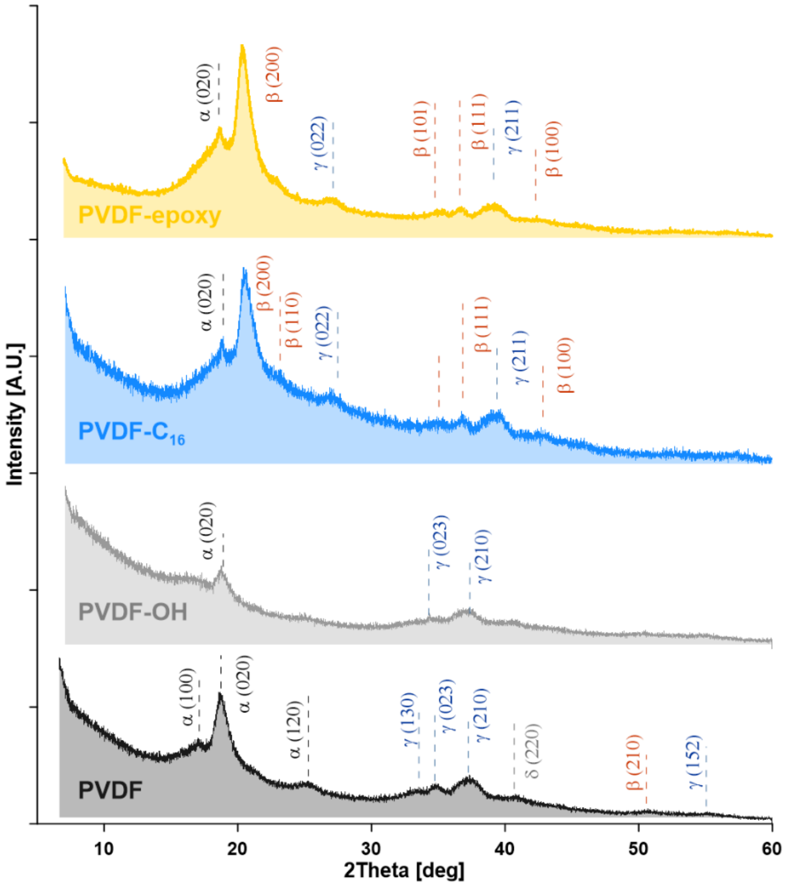

Figure S3. XRD spectra of pristine PVDF membrane, activated PVDF-OH, and modified membranes PVDF-C_{16}, PVDF-epoxy.

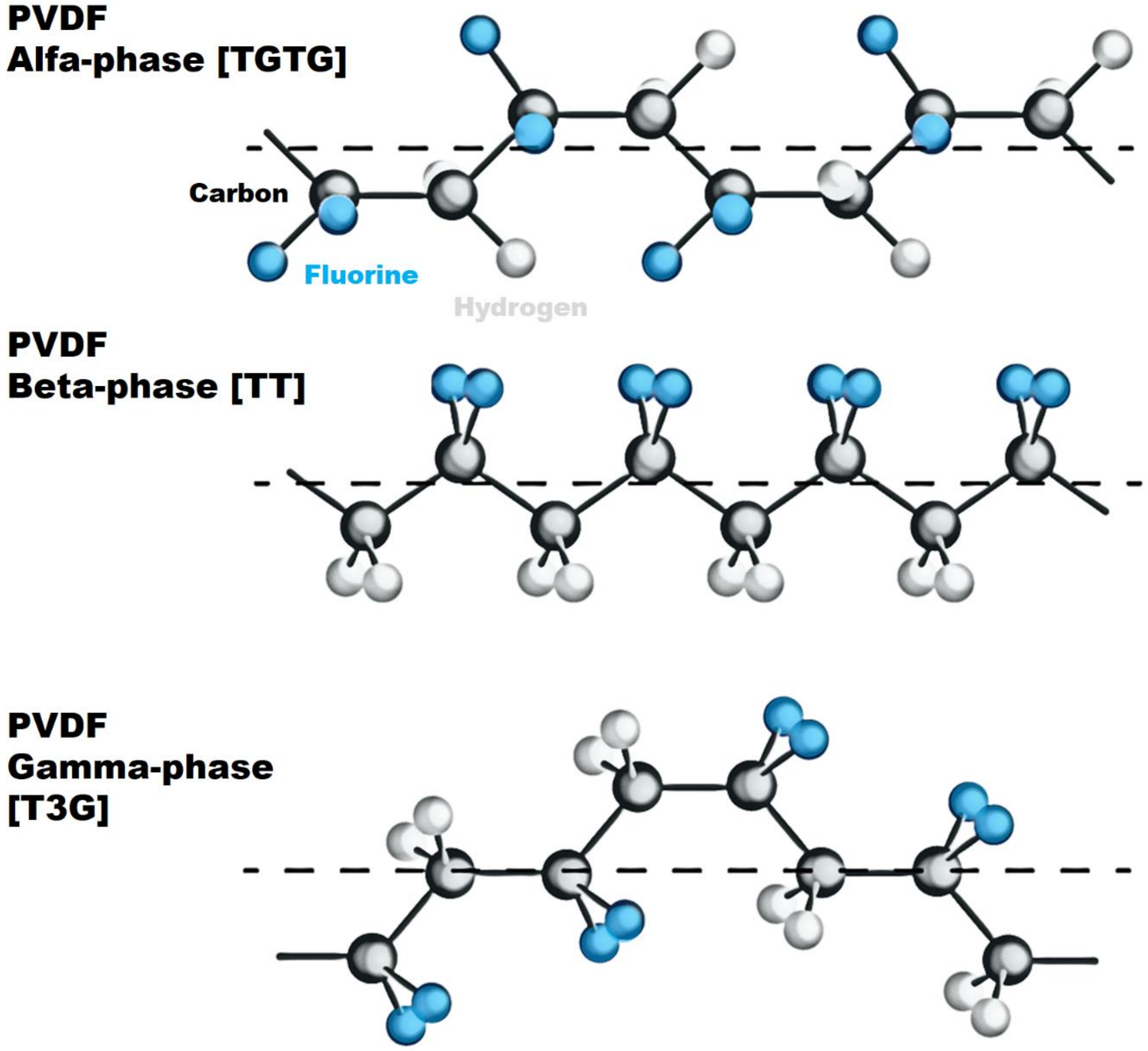

Figure S4. Phases of PVDF.

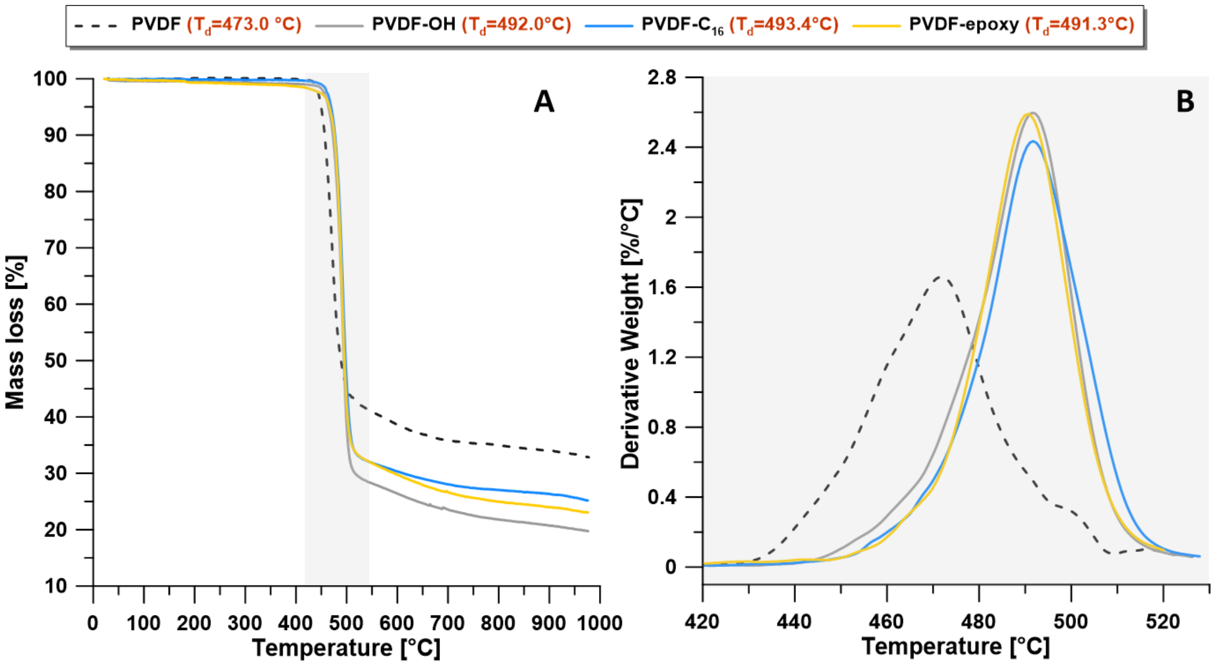

Figure S5. Thermal properties (A - TGA, and B - DTG) of the investigated membranes.

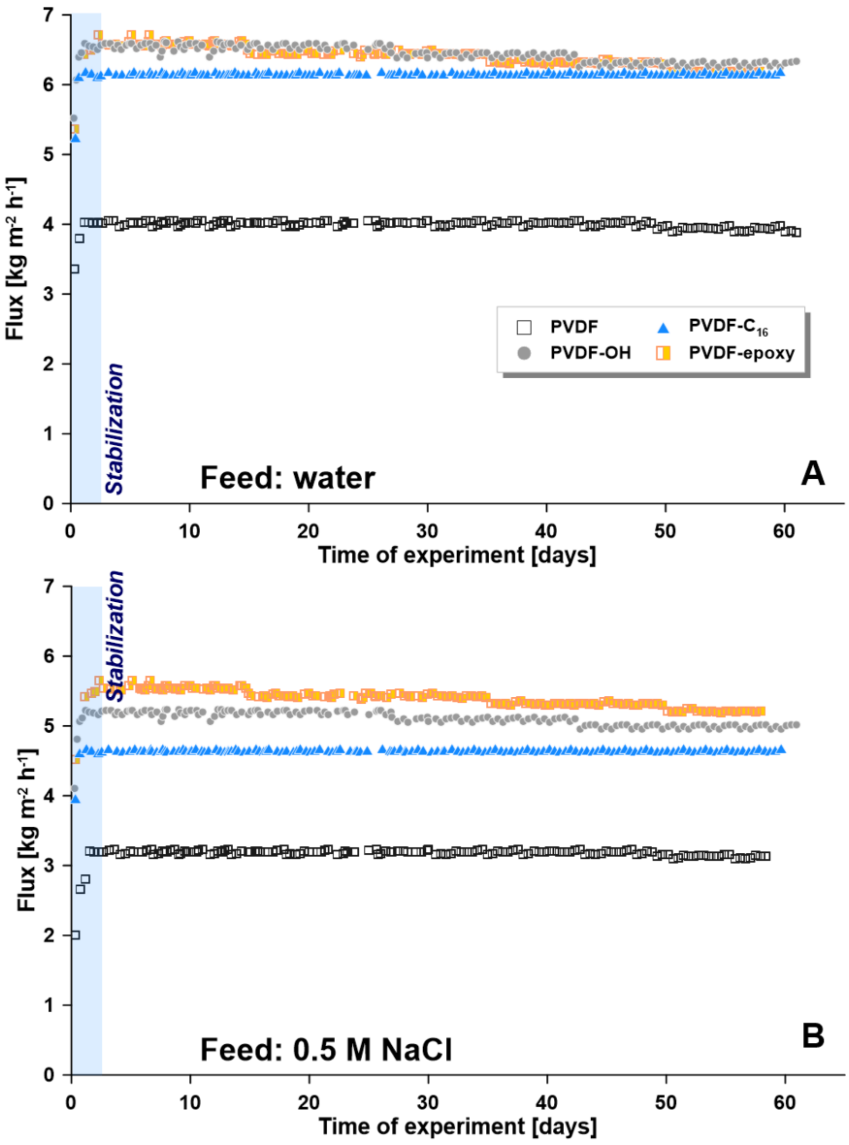

Figure S6. Stability of the membranes during the AGMD process with pure water (A) and 0.5M NaCl (B).

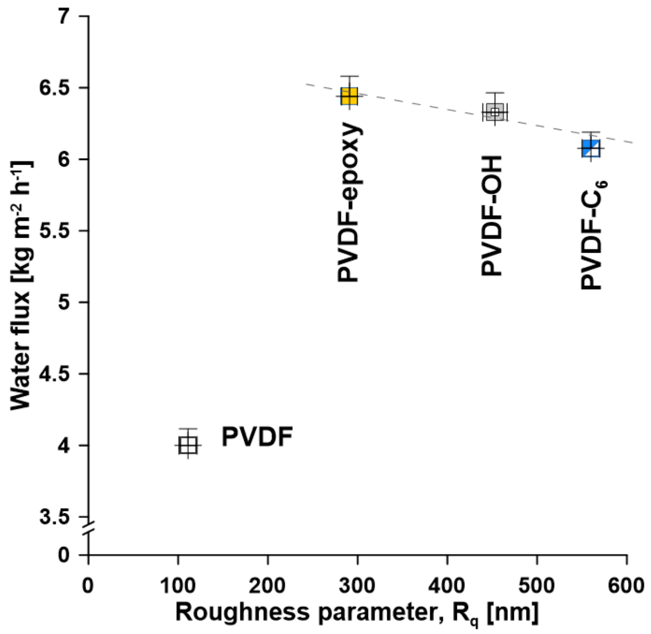

Figure S7. Relation between roughness and transport features of the membranes.

References:

[1] D.Y. Kwok, A.W. Neumann, Contact angle measurement and contact angle interpretation, Adv. Colloid Interf. Sci., 81 (1999) 167-249.

[2] D.K. Owens, R.C. Wendt, Estimation of the surface free energy of polymers, J. Appl. Polym. Sci., 13 (1969) 1741-1747.

[3] E. Chibowski, Surface free energy of a solid from contact angle hysteresis, Adv. Colloid Interface Sci., 103 (2003) 149-172.

[4] I.A. Starostina, N.V. Makhrova, S.O. V., I.V. Aristov, On the Evaluation of the Acidity and Basicity Parameters of the Surface Free Energy of Polymers, J. Adhes., 88 (2012) 751-765.

[5] N. Sato, Y. Shimogaki, Relationship between Surface Free Energy of Underlying Layers and O3-TEOS Chemical Vapor Deposition, ECS J. Solid State Sci. Technol., 2 (2013) 187-190.

[6] T. Rezayi, M.H. Entezari, F. Moosavi, The variation of surface free energy of Al during superhydrophobicity processing, Che. Eng. J., 322 (2017) 181-187.

[7] A. Burdzik, M. Stähler, M. Carmo, D. Stolten, Impact of reference values used for surface free energy determination: An uncertainty analysis, Int. J. Adhes. Adhes., 82 (2018) 1-7.

[8] A. Rudawska, E. Jacniacka, Evaluating uncertainty of surface free energy measurement by the van Oss-Chaudhury-Good method, Int. J. Adhes. Adhes., 82 (2018) 139-145.

[9] M. Shaker, E. Salahinejad, A combined criterion of surface free energy and roughness to predict the wettability of non-ideal low-energy surfaces, Prog. in Org. Coat., 119 (2018) 123-126.

[10] C.M. Guijt, G.W. Meindersma, T. Reith, A.B.d. Haan, Air gap membrane distillation: 2. Model validation and hollow fibre module performance analysis, Sep. Purif. Technol., 43 (2005) 245-255.

[11] A.M. Alklaibi, N. Lior, Transport analysis of air-gap membrane distillation, J. Membr. Sci., 255 (2005) 239-253.

[12] J. Straub, NBS/NRC steam tables. Von L. Haar, J. S. Gallagher und G. S. Kell. Hemisphere Publishing Corp., Washington–New York–London 1984., Chem. Ing. Tech., 57 (1985) 812-812.
[13] L.F. Dumée, H. Alglave, T. Chaffraix, B. Lin, K. Magniez, J. Schütz, Morphology-properties relationship of gas plasma treated hydrophobic meso-porous membranes and their improved performance for desalination by membrane distillation, Appl. Surf. Sci., 363 (2016) 273-285.
[14] R.W. Baker, J.G. Wijmans, Y. Huang, Permeability, permeance and selectivity: A preferred way of reporting pervaporation performance data, J. Membr. Sci., 348 (2010) 346-352.
[15] D.M. Warsinger, J. Swaminathan, E. Guillen-Burrieza, H.A. Arafat, J.H. Lienhard V, Scaling and fouling in membrane distillation for desalination applications: A review, Desalination, 356 (2015) 294-313.
[16] F. Abdulla AlMarzooqi, M. Roil Bilad, H. Ali Arafat, Improving Liquid Entry Pressure of Polyvinylidene Fluoride (PVDF) Membranes by Exploiting the Role of Fabrication Parameters in Vapor-Induced Phase Separation VIPS and Non-Solvent-Induced Phase Separation (NIPS) Processes, Appl. Sci., 7 (2017) 181.
[17] W. Kujawski, P. Adamczak, A. Narebska, A Fully Automated System for the Determination of Pore Size Distribution in Microfiltration and Ultrafiltration Membranes, Sep. Sci. Technol., 24 (1989) 495-506.
